# Associations between hearing difficulty, labor force status, and health burden in young stroke survivors

**DOI:** 10.3389/fstro.2026.1807741

**Published:** 2026-07-24

**Authors:** Elizabeth Tobener, Molly Jacobs, Charles Ellis

**Affiliations:** 1 Department of Speech, Language, and Hearing Sciences, University of Florida, Gainesville, FL, United States; 2 Department of Health Services Research, Management and Policy, University of Florida, Gainesville, FL, United States

**Keywords:** BRFSS, health burden, hearing difficulty, labor status, young stroke patients

## Abstract

**Background:**

Young adults with stroke often experience functional impairments, which may be exacerbated by hearing loss. This study examined associations between employment disruptions and functional, physical, and mental health limitations among young stroke survivors with and without self-reported hearing difficulty.

**Methods:**

This analysis used data from the 2020 to 2023 Behavioral Risk Factor Surveillance System (BRFSS) for adults aged 18–49 with self-reported stroke. Outcome measures included functional limitations—including concentration/memory, mobility/walking, dressing/bathing, and completing errands—and the number of days with poor physical, mental, and overall health. Logistic and negative binomial regressions examined the associations between these outcomes, employment status, and hearing difficulty, controlling for sample heterogeneity. Interactions between hearing difficulty and employment status were assessed to examine potential differential effects.

**Results:**

Respondents who reported hearing difficulty had significantly greater odds of functional limitations, including difficulty concentrating (OR = 2.60, 95% CI: 1.56–4.35), doing errands independently (OR = 3.71, 95% CI: 2.22–6.21), dressing or bathing (OR = 6.09, 95% CI: 3.30–11.24), and walking or climbing stairs (OR = 3.66, 95% CI: 1.89–7.09). Hearing difficulty was also independently associated with a greater number of poor physical health days (IRR = 1.39, 95% CI: 1.02–1.89) and poor mental health days (IRR = 1.67, 95% CI: 1.30–2.14). Being out of work or out of the labor force was independently associated with poorer functional, physical, and mental health outcomes.

**Conclusions:**

These findings underscore the need for integrated post-stroke rehabilitation strategies that address sensory, cognitive, and social dimensions.

## 1 Introduction

Stroke in young adulthood presents a distinct and growing public health challenge, with implications that extend far beyond the acute clinical episode. Unlike older adults, younger individuals who experience a stroke are often in the midst of their most economically and socially productive years. The definition of young stroke is highly variable in the current literature with young stroke being defined with multiple age cutoffs: 40, 45, 50 and up to 65 years (Takeshita et al., 2021). Consequently, stroke-related impairments in this population can have profound and lasting effects on physical function, mental wellbeing, and participation in daily life, including employment and social roles (George et al., 2017; Aarnes et al., 2011). Although advancements in acute stroke care have improved survival rates, many young stroke survivors face persistent difficulties in mobility, mood, cognition, and overall quality of life long after hospital discharge (Ekstrand and Brogårdh, 2022; Maaijwee et al., 2014).

An emerging but underrecognized factor that may influence post-stroke outcomes in this demographic is hearing loss. Hearing loss, which affects an estimated 15% of adults in the United States and often begins in early to mid-adulthood, is associated with increased risk of depression, cognitive decline, and social withdrawal (Lin et al., 2011; Genther et al., 2013). Among stroke survivors, hearing loss may exacerbate communication challenges, hinder engagement in rehabilitation, and intensify emotional distress—yet it remains rarely screened for or addressed in post-stroke care pathways (Koohi et al., 2017). The intersection of stroke and hearing loss is particularly important to consider in younger populations, where the implications for long-term disability, social integration, and economic productivity are especially pronounced.

Despite this potential impact, limited research has systematically explored how hearing status may influence the health trajectories of young adults with stroke. Furthermore, employment status—both as an outcome and as a social determinant of health—remains an underexamined factor in the context of post-stroke recovery in this age group. Return to work is not only an indicator of functional recovery, but also a contributor to improved psychological wellbeing and financial independence (Westerlind et al., 2020; Donker-Cools et al., 2016). However, individuals with stroke and comorbid sensory impairments may face additional barriers to re-employment, compounding the risk of long-term disability.

This study aims to fill this gap by examining the associations between self-reported hearing difficulty, employment, and health-related limitations among young adults with stroke. Specifically, we assess how hearing difficulty correlates with functional, physical, and mental health challenges in this population, while also considering the role of employment as both a protective factor and a potential source of disparity.

## 2 Methods

### 2.1 Data

This study utilized publicly available data from the Behavioral Risk Factor Surveillance System (BRFSS) for 2020, 2021, 2022, and 2023 (Imoisili et al., 2024). The BRFSS is an ongoing, state-based, cross-sectional telephone survey conducted annually by the Centers for Disease Control and Prevention (CDC), collecting data on health-related risk behaviors, chronic health conditions, and use of preventive services among non-institutionalized adults aged 18 years and older in the United States (US) (Erickson et al., 2009). Administered in all 50 states, the District of Columbia, and participating U.S. territories, the BRFSS is the most extensive continuously conducted health survey system in the world. Studies have frequently pooled multiple years of BRFSS data to study national-level health outcomes in the US population (Das Gupta et al., 2023; Kim et al., 2025).

The BRFSS employs a multistage, stratified sampling design that utilizes random digit dialing techniques for both landline and cellular phones. The CDC provided complex survey design variables—including strata, primary sampling unit (PSU), and individual-level sampling weights—to ensure nationally representative estimates and account for non-response bias, oversampling, and disproportionate probabilities of selection.

The BRFSS core questionnaire included modules on sociodemographic characteristics, health status, healthcare access, sensory difficulty, and chronic conditions, including a self-reported history of stroke. Additional questions assessed functional limitations, mental health (e.g., number of poor mental health days in the past 30 days), and physical health (e.g., number of physically unhealthy days). These items are standardized across states, allowing for consistent comparisons across geographic and demographic groups. All BRFSS data are publicly available and de-identified. Therefore, this study was deemed exempt from Institutional Review Board (IRB) approval under federal guidelines for the secondary analysis of public datasets.

### 2.2 Sample

Although no universally accepted age threshold exists, young stroke is most commonly defined as stroke occurring in adults younger than 50 years (Abedi et al., 2023; Bukhari et al., 2023). Therefore, each year of the BRFSS was limited to individuals (1) below age 50, (2) who indicated that they had been told by a doctor, nurse, or other healthcare professional that they had a stroke, (3) provided a “yes” or “no” response when asked if they were deaf or had serious difficulty hearing, and (4) responded to key survey items concerning functional abilities, mental health, and physical health. These criteria resulted in a sample of 6,758 with roughly 25% from each year.

### 2.3 Functional abilities

Four functional health outcomes were examined to assess the extent of disability among stroke survivors. These outcomes were based on self-reported difficulty with core activities of daily living (ADLs) and instrumental activities of daily living (IADLs), as measured in the BRFSS. Specifically, respondents were asked whether they experienced: (1) difficulty concentrating, remembering, or making decisions due to a physical, mental, or emotional condition; (2) difficulty walking or climbing stairs; (3) difficulty dressing or bathing; and (4) difficulty doing errands alone, such as visiting a doctor's office or shopping. Each outcome was treated as a binary variable indicating the presence or absence of the functional limitation. These items provide a multidimensional view of cognitive, mobility, and self-care challenges, offering insight into the everyday functional burdens experienced by stroke survivors across different age groups.

### 2.4 Mental/physical/poor health days

Mental and physical health outcomes were measured using standardized items from the BRFSS that assess the frequency of health-related distress and activity limitation. Respondents were asked: (1) “Now thinking about your mental health, which includes stress, depression, and problems with emotions, for how many days during the past 30 days was your mental health not good?”; (2) “Now thinking about your physical health, which includes physical illness and injury, for how many days during the past 30 days was your physical health not good?”; and (3) “During the past 30 days, how many days did poor physical or mental health keep you from doing your usual activities, such as self-care, work, or recreation?” Responses to each item ranged from 0 to 30 days, with higher values indicating a greater number of days with poor health or activity limitation. These variables were analyzed as continuous outcomes to reflect the burden of impaired wellbeing and functioning. Together, they provide a subjective yet meaningful measure of recent health-related quality of life, capturing distinct but interrelated dimensions of mental distress, physical illness, and the extent to which these issues interfere with daily life.

### 2.5 Demographic characteristics

Demographic characteristics were collected using standardized survey items. Respondents reported age in years and sex was coded by interviewers as either male or female based on vocal cues, including the respondent's name and tone of voice. If the interviewer was unable to determine the respondent's sex with confidence, the respondent was asked directly, “I'm required to ask, are you male or female?”

Race and ethnicity were collected using a two-step process. Respondents were first asked whether they identified as Hispanic, Latino/a, or of Spanish origin. They were then asked to select one or more racial categories: White, Black or African American, American Indian or Alaska Native, Asian, Native Hawaiian or Other Pacific Islander, or other. In the final public-use dataset, responses are recoded into a single race/ethnicity variable using standardized CDC protocols, prioritizing Hispanic ethnicity and single-race categories for consistency in reporting. The national sample consisted of only 2.83% Non-Hispanic Asian, 1.60% American Indian/Alaskan Native, Non-Hispanic, and 3.70% Non-Hispanic Other race. These groups had even smaller representation among stroke survivors; thus, they were combined into a single cohort.

Marital status was reported as married, divorced, widowed, separated, never married, or a member of an unmarried couple; however, these categories were collapsed into two main categories: married and not married. Educational attainment was determined by asking the respondent about the highest grade or year of school they had completed. Response options included less than a high school education, a high school graduate or GED, some college or technical school, and a college graduate. These were coded as less than high school, high school, or some college, and college or above.

All respondents indicated whether they were insured during the interview and selected the category appropriate to their annual pre-tax household income. BRFSS categories included less than $10,000, $10,000 to less than $15,000, $15,000 to less than $20,000, $20,000 to less than $25,000, $25,000 to less than $35,000, $35,000 to less than $50,000, $50,000 to less than $75,000, and $75,000 or more. In this study, household income was collapsed into three groups: less than $35,000, $35,000 to less than $100,000, and $100,000 and above. These thresholds align with those used in prior research analyzing BRFSS data (Silver et al., 2025). Income below $35,000 corresponds to households below approximately 250% to 300% of the federal poverty level, which many government programs use to determine eligibility and insurance under the Affordable Care Act (US Department of Health and Human Services, 2025). The $35,000 to less than $100,000 range represents middle-income groups, and the $100,000 + category reflects higher-income respondents (US Department of Health and Human Services, 2025; SHADAC, 2025). The $100,000 threshold also aligns with the upper bound used in income imputation strategies for BRFSS analysis, facilitating comparability with similar public health studies (US Department of Health and Human Services, 2025; SHADAC, 2025).

Respondents reported their employment status as employed for wages, self-employed, out of work for 1 year or more, out of work for less than 1 year, a homemaker, a student, or retired. These categories were collapsed into employed (including working for wages and self-employed), unemployed (including out of work for ≥ 1 year and out of work for <1 year), and out of the labor force (including homemakers, students, and retirees).

### 2.6 Statistical analysis

Statistical analyses were conducted to examine differences in functional, physical, and mental health outcomes between young stroke survivors with and without self-reported hearing difficulty. Logistic regression models were used to estimate associations between hearing difficulty and each of the four binary functional outcomes, including difficulty concentrating or remembering, difficulty walking or climbing stairs, difficulty dressing or bathing, and difficulty completing errands independently.

For count outcomes representing the number of poor physical health days, poor mental health days, and days in which poor physical or mental health limited usual activities during the past 30 days, survey-weighted negative binomial regression models were estimated. Preliminary analyses indicated substantial overdispersion in these outcomes, and likelihood ratio tests strongly rejected the assumption of equidispersion required for Poisson regression (*p* < 0.001). Therefore, negative binomial regression was selected as the preferred modeling approach.

The primary independent variable of interest was self-reported hearing difficulty (yes/no). All models adjusted for age, sex, race/ethnicity, marital status, educational attainment, household income, insurance coverage, and employment status (employed, unemployed, or out of the labor force), with employed respondents serving as the reference group.

Since hearing difficulty may be associated with employment opportunities and workforce participation through communication challenges and related functional limitations, an interaction term between hearing difficulty and employment status was included in all models. This interaction was intended to evaluate whether the association between employment status and health outcomes differed among young stroke survivors with hearing loss. Due to concerns regarding sparse subgroups when estimating interaction effects, subgroup sample sizes for the hearing loss and employment status categories are presented in Appendix Table 1. Potential multicollinearity among independent variables, including the hearing loss and employment status interaction terms, was assessed using variance inflation factors (VIFs). No evidence of problematic multicollinearity was observed (mean VIF = 1.53; maximum VIF = 2.66).

All analyses accounted for the complex sampling design of the BRFSS. Survey strata, primary sampling units, and final sampling weights provided by the Centers for Disease Control and Prevention were incorporated using Stata's survey estimation procedures (svy). Variance estimates were computed using Taylor-series linearization to obtain nationally representative estimates and appropriate standard errors. As part of model diagnostics, multicollinearity was assessed using variance inflation factors (VIFs). No evidence of problematic multicollinearity was identified (mean VIF = 1.53; maximum VIF = 2.66). Statistical significance was evaluated at α = 0.05. All analyses were conducted using Stata version 18 (StataCorp, College Station, TX).

## 3 Results

### 3.1 Descriptive characteristics

Table 1 shows the characteristics of the full sample (*N* = 6,768), stratified by self-reported hearing status. Approximately 10.35% of respondents reported being deaf or having serious difficulty hearing (*n* = 715), while 89.65% reported no difficulty (*n* = 6,053). Respondents with hearing difficulty reported significantly more days of poor physical health (mean = 14.57, SD = 12.55 vs. 9.23, SD = 11.56; F = 112.88, *p* < 0.0001), poor mental health (15.24, SD = 12.67 vs. 10.24, SD = 11.66; *F* = 96.68, *p* < 0.0001), and combined poor health days (14.24, SD = 12.38 vs. 9.87, SD = 11.38; *F* = 69.43, *p* < 0.0001) compared to those without hearing difficulty. Statistically significant differences were found across most demographic variables. Those with hearing difficulty were more likely to be male (52.6% vs. 41.9%; χ^2^ = 30.00, *p* < 0.0001), less likely to be White (55.1% vs. 60.1%; χ^2^ = 43.82, *p* < 0.0001), and more likely to identify as Hispanic or “Other” race/ethnicity. They were also more likely to have lower educational attainment, with 8.3% having less than a high school education compared to 3.3% in the group without hearing difficulty (χ^2^ = 76.18, *p* < 0.0001), and were less likely to be married (28.3% vs. 37.7%; χ^2^ = 24.74, *p* < 0.0001). Nearly 70% of those with hearing difficulty had annual household incomes below $35,000, compared to 51.6% of those without (χ^2^ = 58.02, *p* < 0.0001). They were more likely to be uninsured (15.6% vs. 11.6%; χ^2^ = 9.19, *p* = 0.00) and to have public rather than private insurance coverage. Functional limitations were significantly more prevalent among individuals with hearing difficulty, including higher rates of difficulty with concentration (62.78% vs. 36.71%; χ^2^ = 181.24, *p* < 0.0001), mobility (59.3% vs. 31.53%; χ^2^ = 216.73, *p* < 0.0001), personal care (32.11% vs. 13.46%; χ^2^ = 169.26, *p* < 0.0001), and errands (47.6% vs. 24.95%; χ^2^ = 163.56, *p* < 0.0001). Self-rated health status was also lower, with 63.8% of those with hearing difficulty reporting fair or poor health compared to 43.7% of those without (χ^2^ = 122.04, *p* < 0.0001).

**Table 1:** Sample characteristics.

Characteristic	Full sample (*N* = 6,768)		No difficulty hearing (*N* = 6,053, 89.65%)		Deaf or serious difficulty hearing (*N* = 715, 10.35%)			
	Mean	Std dev	Mean	Std dev	Mean	Std dev	*F*	Prob
Age (18–49)	39.79	7.79	39.75	7.78	40.09	7.87	1.15	0.28
Days poor physical health (0–30)	9.78	11.78	9.23	11.56	14.57	12.55	112.88	<0.00
Days poor mental health (0–30)	10.76	11.86	10.24	11.66	15.24	12.67	96.6	<0.00
Days poor physical health or mental health (0–30)	10.38	11.58	9.87	11.38	14.24	12.38	69.4	<0.00
	***N***	**PCT**	***N***	**PCT**	***N***	**PCT**	*χ^2^*	**Prob**
Male	2,910	43	2,534	41.86	376	52.59	30.00	<0.0001
Female	3,858	57	3,519	58.14	339	47.41		
White	4,033	59.59	3,639	60.12	394	55.1	43.82	<0.0001
Black	875	12.93	818	13.51	57	7.97		
Other	980	14.48	841	13.89	139	19.44		
Hispanic	880	13	755	12.47	125	17.48		
Less than high school	259	3.85	200	3.32	59	8.32	76.18	<0.0001
High school, some college	4,171	61.94	3,748	62.2	423	59.66		
College or above	1,670	24.8	1,540	25.56	130	18.34		
Not Married	4,282	63.27	3,769	62.27	513	71.75	24.74	<0.0001
Married	2,486	36.73	2,284	37.73	202	28.25		
Employed	3,375	70.59	3,114	71.47	261	61.56	18.63	<0.0001
Out of work	750	15.69	660	15.15	90	21.23		
OOLF	656	13.72	583	13.38	73	17.22		
Less than $35,000	3,049	53.24	2,645	51.6	404	67.22	58.02	<0.0001
$35,000 to <$100,000	1,970	34.4	1,809	35.29	161	26.79		
$100,000 or more	708	12.36	672	13.11	36	5.99		
No health plan	788	12.03	681	11.62	107	15.6	9.19	0.0024
Health plan	5,760	87.97	5,181	88.38	579	84.4		
Private insurance	1,932	37.33	1,820	39.28	112	20.66	71.91	<0.0001
Public insurance	2,683	51.85	2,328	50.25	355	65.5		
No insurance	560	10.82	485	10.47	75	13.84		
Excellent, very good, good	3,654	54.22	3,397	56.34	257	36.2	122.04	<0.0001
Fair, poor	3,085	45.78	2,632	43.66	453	63.8		
No difficulty concentrating, remembering	4,081	60.54	3,816	63.29	265	37.22	181.24	<0.0001
Difficulty concentrating, remembering	2,660	39.46	2,213	36.71	447	62.78		
No difficulty doing errands alone	4,866	72.66	4,495	75.05	371	52.4	163.56	<0.0001
Difficulty doing errands alone	1,831	27.34	1,494	24.95	337	47.6		
No difficulty dressing or bathing	5,677	84.57	5,195	86.54	482	67.89	169.26	<0.0001
Difficulty dressing or bathing	1,036	15.43	808	13.46	228	32.11		
No difficulty walking or climbing stairs	4,404	65.54	4,115	68.47	289	40.7	216.73	<0.0001
Difficulty walking or climbing stairs	2,316	34.46	1,895	31.53	421	59.3		

### 3.2 Functional abilities

Multivariable logistic regression models identified several factors associated with functional limitations among young adults with stroke (Table 2). Across all four outcomes, hearing difficulty emerged as one of the strongest and most consistent predictors of functional impairment. Respondents with hearing difficulty had significantly greater odds of reporting difficulty concentrating, remembering, or making decisions (OR = 2.60, 95% CI: 1.56, 4.35), difficulty doing errands independently (OR = 3.71, 95% CI: 2.22, 6.21), difficulty walking or climbing stairs (OR = 3.66, 95% CI: 1.89, 7.09), and difficulty dressing or bathing (OR = 6.09, 95% CI: 3.30, 11.24) compared with respondents without hearing difficulty.

**Table 2:** Characteristics associated with the likelihood of functional difficulty among respondents with young stroke.

Characteristic	Odds ratio	*t*–stat	*p*–value	95% confidence interval	
Difficulty concentrating, remembering, or making decisions					
Intercept	**0.28**	−4.06	0.00	0.15	0.52
Age	1.00	−0.47	0.64	0.98	1.01
Income $35,000 to <$100,000	1.27	1.67	0.10	0.96	1.67
Income less than $35,000	0.77	−1.59	0.11	0.57	1.06
Black	**0.62**	−3.27	0.00	0.47	0.83
Hispanic	0.86	−0.96	0.34	0.64	1.17
Other	0.98	−0.09	0.93	0.69	1.40
High school, some college	**2.33**	4.25	0.00	1.58	3.45
Less than high school	**1.36**	2.11	0.03	1.02	1.80
Female	**1.35**	2.74	0.01	1.09	1.67
Married	**0.66**	−3.39	0.00	0.51	0.84
No Insurance	1.18	0.98	0.33	0.85	1.64
Public insurance	1.01	0.07	0.94	0.74	1.38
OOLF	**1.85**	3.60	0.00	1.32	2.58
Out of work	**2.40**	6.57	0.00	1.85	3.11
Deaf/hard of hearing	**2.60**	3.65	0.00	1.56	4.35
OOLF^*^deaf/hard of hearing	0.40	−1.58	0.11	0.13	1.24
Out of work^*^deaf/hard of hearing	1.09	0.25	0.81	0.54	2.20
**Difficulty doing errands alone such as visiting a doctor's office or shopping**					
Intercept	**0.07**	−7.91	0.00	0.04	0.13
Age	1.00	0.47	0.64	0.99	1.02
Income $35,000 to <$100,000	1.32	1.77	0.08	0.97	1.78
Income less than $35,000	0.88	−0.75	0.46	0.64	1.22
Black	0.74	−1.92	0.06	0.54	1.01
Hispanic	0.96	−0.23	0.82	0.68	1.35
Other	1.21	1.17	0.24	0.88	1.67
High school, some college	1.19	0.84	0.40	0.79	1.79
Less than high school	1.11	0.63	0.53	0.81	1.52
Female	0.96	−0.33	0.74	0.76	1.21
Married	**0.60**	−3.55	0.00	0.45	0.79
No insurance	**1.53**	2.20	0.03	1.05	2.22
Public insurance	1.15	0.78	0.43	0.81	1.66
OOLF	**2.50**	4.88	0.00	1.73	3.62
Out of work	**6.90**	12.68	0.00	5.12	9.31
Deaf/hard of hearing	**3.71**	4.99	0.00	2.22	6.21
OOLF^*^deaf/hard of hearing	1.07	0.11	0.91	0.33	3.43
Out of work^*^deaf/hard of hearing	0.75	−0.83	0.41	0.37	1.49
**Difficulty dressing or bathing**					
Intercept	**0.02**	−9.05	0.00	0.01	0.05
Age	1.02	1.80	0.07	1.00	1.03
Income $35,000 to <$100,000	1.37	1.65	0.10	0.94	2.00
Income less than $35,000	0.79	−1.01	0.31	0.50	1.25
Black	1.21	1.04	0.30	0.84	1.73
Hispanic	1.60	2.28	0.02	1.07	2.41
Other	1.07	0.29	0.77	0.70	1.62
High school, some college	**1.17**	0.67	0.50	0.74	1.87
Less than high school	**0.92**	−0.41	0.68	0.62	1.36
Female	0.77	−1.80	0.07	0.58	1.02
Married	**0.71**	−2.01	0.05	0.51	0.99
No insurance	1.07	0.27	0.79	0.65	1.76
Public insurance	0.82	−0.85	0.40	0.51	1.31
OOLF	**2.49**	3.38	0.00	1.47	4.24
Out of work	8.47	9.66	0.00	5.49	13.06
Deaf/hard of hearing	**6.09**	5.77	0.00	3.30	11.24
OOLF^*^deaf/hard of hearing	**0.32**	−2.09	0.04	0.11	0.93
Out of work^*^deaf/hard of hearing	**0.44**	−2.13	0.03	0.21	0.94
**Difficulty walking or climbing stairs**					
Intercept	**0.04**	−8.00	0.00	0.02	0.09
Age	**1.03**	3.87	0.00	1.01	1.05
Income $35,000 to <$100,000	**1.60**	3.01	0.00	1.18	2.17
Income less than $35,000	0.91	−0.55	0.58	0.64	1.29
Black	1.01	0.07	0.94	0.75	1.37
Hispanic	1.01	0.07	0.95	0.71	1.44
Other	1.28	1.16	0.25	0.84	1.95
High school, some college	1.29	1.10	0.27	0.82	2.02
Less than high school	1.04	0.23	0.82	0.73	1.49
Female	1.05	0.37	0.71	0.83	1.32
Married	**0.64**	−3.37	0.00	0.50	0.83
No insurance	**1.62**	2.59	0.01	1.13	2.33
Public insurance	1.22	1.09	0.28	0.85	1.75
OOLF	1.33	1.64	0.10	0.94	1.88
Out of work	**4.24**	9.99	0.00	3.19	5.63
Deaf/hard of hearing	**3.66**	3.85	0.00	1.89	7.09
OOLF^*^deaf/hard of hearing	1.67	0.79	0.43	0.47	5.90
Out of work^*^deaf/hard of hearing	1.25	0.54	0.59	0.55	2.85

Reference group: income (≥$1,00,000), race/ethnicity (non–hispanic, white), education (college and above), sex (male), marital status (not married), insurance (private insurance), employment status (employed), hearing (no difficulty hearing). **Indicates** significance at a 95% confidence level. ^*^ denotes an interaction.

Employment status was also strongly associated with functional outcomes. Compared with employed respondents, those who were out of work or out of the labor force generally exhibited greater odds of mobility, self-care, and instrumental activity limitations. Married respondents consistently reported lower odds of several functional limitations, including difficulty concentrating and difficulty completing errands independently. Evidence for interaction between hearing difficulty and employment status was limited. Although significant interactions were observed for selected outcomes, the magnitude of these effects was considerably smaller than the main effects associated with hearing difficulty itself. Overall, the findings suggest that hearing difficulty and labor-force disengagement independently contribute to functional limitations among young stroke survivors.

### 3.3 Healthy days

Table 3 presents factors associated with the number of poor physical health days reported during the past 30 days. Hearing difficulty was independently associated with greater physical health burden, with respondents reporting approximately 39% more poor physical health days than those without hearing difficulty (IRR = 1.39, 95% CI: 1.02, 1.89). Employment status was also strongly associated with physical health. Compared with employed respondents, those who were out of work reported approximately 80% more poor physical health days (IRR = 1.80, 95% CI: 1.56, 2.07). Black respondents reported fewer poor physical health days than non-Hispanic White respondents, while higher household income was generally associated with fewer poor physical health days. A significant interaction between hearing difficulty and being out of the labor force was observed (IRR = 1.89, 95% CI: 1.18, 3.03), suggesting that the combined presence of hearing difficulty and labor-force disengagement was associated with an elevated physical health burden.

**Table 3:** Characteristics associated with days of reported poor physical health among respondents with young stroke.

Characteristic	IRR	*t*–stat	*p*–value	95% confidence interval	
Intercept	**2.59**	4.43	0.00	1.70	3.95
Age	**1.01**	3.23	0.00	1.01	1.02
Income $35,000 to <$100,000	**1.21**	2.16	0.03	1.02	1.44
Income less than $35,000	0.86	−1.60	0.11	0.72	1.03
Black	**0.74**	−3.36	0.00	0.63	0.88
Hispanic	1.07	0.82	0.41	0.91	1.26
Other	1.19	1.90	0.06	0.99	1.42
High school, some college	1.11	0.93	0.35	0.89	1.39
Less than high school	**1.18**	2.02	0.04	1.00	1.38
Female	**1.16**	2.32	0.02	1.02	1.31
Married	0.93	−1.06	0.29	0.80	1.07
No insurance	**1.28**	2.82	0.01	1.08	1.53
Public insurance	1.16	1.61	0.11	0.97	1.38
OOLF	1.18	1.75	0.08	0.98	1.42
Out of work	**1.80**	8.14	0.00	1.56	2.07
Deaf/hard of hearing	**1.39**	2.10	0.04	1.02	1.89
OOLF^*^deaf/hard of hearing	**1.89**	2.65	0.01	1.18	3.03
Out of work^*^deaf/hard of hearing	0.81	−1.08	0.28	0.56	1.18

Dependent variable: days physical health not good (0–30). Reference group: income (≥$100,000), race/ethnicity (non–hispanic, white), education (college and above), sex (male), marital status (not married), insurance (private insurance), employment status (employed), hearing (no difficulty hearing). **Indicates** significance at a 95% confidence level. ^*^ denotes an interaction.

Table 4 presents factors associated with the number of poor mental health days reported during the past 30 days. Respondents with hearing difficulty experienced substantially greater mental health burden than those without hearing difficulty, reporting approximately 67% more poor mental health days (IRR = 1.67, 95% CI: 1.30, 2.14). Married respondents reported fewer poor mental health days than unmarried respondents, while lower household income and labor-force disengagement were associated with worse mental health outcomes. Although hearing difficulty and employment status were independently associated with poor mental health days, the interaction terms were not statistically significant, suggesting that the effect of hearing difficulty on mental health burden was relatively consistent across employment groups.

**Table 4:** Characteristics associated with days of reported poor mental health among respondents with young stroke.

Characteristic	IRR	*t*–stat	*p*–value	95% confidence interval	
Intercept	**8.83**	12.99	0.00	6.36	12.27
Age	**0.99**	−2.14	0.03	0.99	1.00
Income $35,000 to <$100,000	**1.20**	2.38	0.02	1.03	1.40
Income less than $35,000	1.02	0.26	0.80	0.86	1.22
Black	**0.78**	−3.09	0.00	0.67	0.92
Hispanic	0.85	−1.57	0.12	0.69	1.04
Other	1.10	0.98	0.33	0.91	1.31
High school, some college	**1.22**	1.92	0.05	1.00	1.50
Less than high school	**1.21**	2.41	0.02	1.04	1.40
Female	**1.23**	3.19	0.00	1.08	1.39
Married	**0.77**	−3.78	0.00	0.67	0.88
No insurance	1.17	1.76	0.08	0.98	1.40
Public insurance	1.03	0.29	0.77	0.85	1.24
OOLF	**1.41**	3.37	0.00	1.15	1.72
Out of work	**1.28**	2.96	0.00	1.09	1.51
Deaf/hard of hearing	**1.67**	4.01	0.00	1.30	2.14
OOLF^*^deaf/hard of hearing	0.87	−0.63	0.53	0.56	1.34
Out of work^*^deaf/hard of hearing	0.79	−1.46	0.14	0.58	1.08

Dependent variable: days mental health not good (0–30). Reference group: income (≥$100,000), race/ethnicity (non–hispanic, white), education (college and above), sex (male), marital status (not married), insurance (private insurance), employment status (employed), hearing (no difficulty hearing). **Indicates** significance at a 95% confidence level. ^*^ denotes an interaction.

Table 5 presents factors associated with the number of days during which poor physical or mental health limited usual activities. Hearing difficulty remained significantly associated with greater health burden, while respondents who were out of work experienced more than twice the rate of poor health days compared with employed respondents (IRR = 2.27, 95% CI: 1.93, 2.66). Married respondents reported fewer poor health days than unmarried respondents, and Black respondents reported fewer poor health days than non-Hispanic White respondents. Neither interaction term reached statistical significance, indicating that hearing difficulty and employment status primarily exerted independent rather than synergistic effects on overall health burden.

**Table 5:** Characteristics associated with days of reported poor physical or mental health among respondents with young stroke.

Characteristic	IRR	*t*–stat	*p*–value	95% confidence interval	
Intercept	**3.85**	6.66	0.00	2.59	5.72
Age	**1.01**	1.97	0.05	1.00	1.02
Income $35,000 to <$100,000	1.11	1.21	0.23	0.94	1.31
Income less than $35,000	0.92	−0.85	0.40	0.75	1.12
Black	0.88	−1.33	0.18	0.74	1.06
Hispanic	1.15	1.42	0.16	0.95	1.39
Other	1.25	1.73	0.08	0.97	1.61
High school, some college	1.13	1.08	0.28	0.91	1.41
Less than high school	0.96	−0.40	0.69	0.80	1.15
Female	0.98	−0.30	0.77	0.86	1.12
Married	**0.77**	−3.63	0.00	0.67	0.89
No insurance	**1.23**	2.16	0.03	1.02	1.48
Public insurance	1.20	1.81	0.07	0.98	1.47
OOLF	**1.78**	5.92	0.00	1.47	2.15
Out of work	**2.27**	10.07	0.00	1.93	2.66
Deaf/hard of hearing	1.32	1.37	0.17	0.89	1.95
OOLF^*^deaf/hard of hearing	1.26	0.81	0.42	0.72	2.20
Out of work^*^deaf/hard of hearing	1.02	0.09	0.93	0.67	1.54

Dependent variable: days physical or mental health not good (0–30). Reference group: income (≥$1,00,000), race/ethnicity (non–hispanic, white), education (college and above), sex (male), marital status (not married), insurance (private insurance), employment status (employed), hearing (no difficulty hearing). **Indicates** significance at a 95% confidence level. ^*^ denotes an interaction.

**Figure 1:**
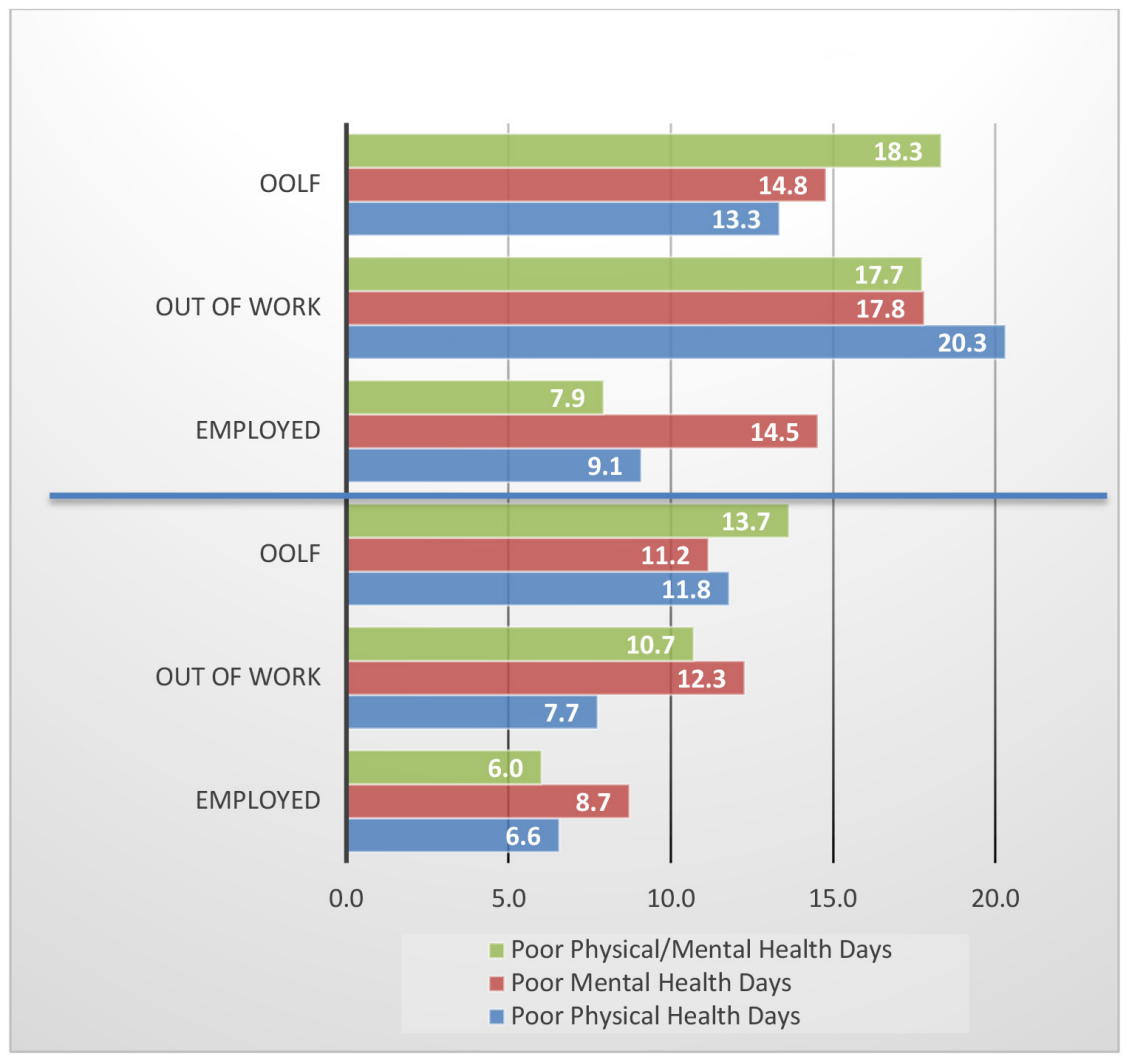
Predicted poor health days.

Adjusted predicted numbers of days of poor physical, mental, and physical/mental health are presented in Figure 1. These predictions demonstrate the highest burden among respondents with hearing difficulty who were out of work or out of the labor force.

## 4 Discussion

This study examined the intersection of self-reported hearing difficulty, stroke, and employment status in adults younger than 50 years, a population whose post-stroke recovery remains underexplored (Keating et al., 2021). The findings indicate that hearing difficulty is associated with poorer functional outcomes, greater physical and mental health burden, and socioeconomic disadvantage among young stroke survivors. Individuals reporting hearing difficulty were more likely to experience limitations in concentration, mobility, and activities of daily living, while also reporting a greater number of poor physical and mental health days. Collectively, these findings suggest that hearing-related challenges may represent an underrecognized factor affecting recovery and long-term functioning after stroke.

Employment status further influenced health outcomes. Stroke survivors who were unemployed or out of the labor force reported the highest burden of poor physical and mental health days, particularly when hearing difficulty was also present. Employment provides financial stability, social engagement, and purpose, all of which are important determinants of health (Hergenrather et al., 2015). Communication difficulties, reduced functional capacity, and other stroke-related impairments may create additional obstacles to workforce participation, potentially contributing to poorer health and quality of life.

These findings are consistent with previous research demonstrating the importance of employment for psychological wellbeing and successful community reintegration after stroke (Westerlind et al., 2020; Donker-Cools et al., 2016). The present results extend this literature by suggesting that hearing difficulty may represent an additional barrier to recovery. Young stroke survivors with hearing difficulty appear to face compounded challenges that may place them at greater risk for long-term disability and reduced participation.

Several sociodemographic disparities were also observed. Participants with hearing difficulty were more likely to be male, have lower socioeconomic status, lack health insurance, and have lower educational attainment. These characteristics align with broader evidence linking social and economic disadvantage to poorer health outcomes and reduced access to care (Mielck et al., 2014). The co-occurrence of hearing difficulty, limited financial resources, and reduced healthcare access highlights the importance of addressing both clinical and social factors during post-stroke rehabilitation (Genther et al., 2013).

The greater prevalence of limitations in mobility, cognition, and self-care among participants with hearing difficulty warrants particular attention. Although the mechanisms underlying these associations cannot be determined from the present study, hearing-related communication challenges may further complicate recovery and daily functioning following stroke. The combination of hearing difficulty and workforce disengagement was also associated with substantially poorer health, underscoring the need for rehabilitation approaches that address sensory, physical, psychological, and social dimensions of recovery.

These findings suggest that hearing difficulty may be overlooked in post-stroke care despite its association with functional and health-related outcomes. Routine hearing screening and referral for audiologic evaluation may help identify modifiable barriers to recovery. In addition, vocational rehabilitation programs that account for hearing-related communication needs may support workforce re-entry and improve long-term participation among young stroke survivors.

### 4.1 Limitations

This study has several limitations. First, BRFSS is a cross-sectional, self-report survey and is therefore subject to recall, reporting, and social desirability biases. Because the data are cross-sectional, the observed relationships should be interpreted as associations rather than evidence of causality. In addition, reverse causality cannot be ruled out; poorer health or functional status may contribute to self-reported hearing difficulty rather than hearing difficulty leading to poorer post-stroke outcomes.

Second, BRFSS does not collect detailed clinical information regarding stroke severity, age at stroke onset, time since stroke, rehabilitation utilization, or comprehensive comorbidity profiles. As a result, residual confounding may be present. In addition, age at interview was used to classify respondents into younger and older stroke groups, and some individuals categorized as older stroke survivors may have experienced their stroke before age 50.

Third, several racial and ethnic groups were combined into an “Other” category because of small sample sizes. This approach preserved sample size but limited the ability to examine differences among specific racial and ethnic populations.

Finally, BRFSS does not include objective audiometric assessments. Hearing status was determined using self-reported hearing difficulty, which may not accurately reflect the presence or severity of hearing loss. Future studies incorporating clinical measures of hearing and more detailed stroke-related variables are needed to better characterize the relationship between hearing difficulty and post-stroke outcomes in young adults.

## 5 Conclusion

Young stroke survivors with self-reported hearing difficulty experienced greater functional limitations and poorer physical and mental health outcomes, particularly when not participating in the workforce. These findings highlight the potential value of incorporating hearing assessment into post-stroke care and support the need for rehabilitation approaches that address both sensory and functional recovery.

## Data Availability

Publicly available datasets were analyzed in this study. This data can be found here: https://www.cdc.gov/brfss/annual_data/annual_2024.html.

## Appendix

**Table A1:** Subgroup sample.

Hearing	Employment	*N*
No hearing difficulty	Employed	3,114
No hearing difficulty	Out of work	660
No hearing difficulty	OOLF	2,279
Hearing difficulty	Employed	261
Hearing difficulty	Out of work	90
Hearing difficulty	OOLF	364
